# Estimation of Pulmonary Arterial Wave Reflection by Echo-Doppler: A Preliminary Study in Dogs With Experimentally-Induced Acute Pulmonary Embolism

**DOI:** 10.3389/fphys.2021.752550

**Published:** 2021-12-08

**Authors:** Tomohiko Yoshida, Tokuhisa Uejima, Syunta Komeda, Katsuhiro Matsuura, Akiko Uemura, Hiromasa Hayama, Takeshi Yamashita, Zeki Yilmaz, Ryou Tanaka

**Affiliations:** ^1^Department of Veterinary Surgery, Tokyo University of Agriculture and Technology, Fuchu, Japan; ^2^The Cardiovascular Institute, Tokyo, Japan; ^3^Department of Cardiology, National Center for Global Health and Medicine, Tokyo, Japan; ^4^Department of Veterinary Internal Medicine, Bursa Uludag University, Bursa, Turkey

**Keywords:** pulmonary hypertension, wave separation analysis, wave reflection, Doppler echocardiography, wave intensity analysis

## Abstract

**Background:** Pulmonary arterial (PA) wave reflection provides additional information for assessing right ventricular afterload, but its applications is hampered by the need for invasive pressure and flow measurements. We tested the hypothesis that PA pressure and flow waveforms estimated by Doppler echocardiography could be used to quantify PA wave reflection.

**Methods:** Doppler echocardiographic images of tricuspid regurgitation and right ventricular outflow tract flow used to estimate PA pressure and flow waveforms were acquired simultaneously with direct measurements with a dual sensor-tipped catheter under various hemodynamic conditions in a canine model of pulmonary hypertension (*n* = 8). Wave separation analysis was performed on echo-Doppler derived as well as catheter derived waveforms to separate PA pressure into forward (Pf) and backward (Pb) pressures and derive wave reflection coefficient (RC) defined as the ratio of peak Pb to peak Pf.

**Results:** Wave reflection indices by echo-Doppler agreed well with corresponding indices by catheter (Pb: mean difference = 0.4 mmHg, 95% limits of agreement = −4.3 to 5.0 mmHg; RC: bias = 0.13, 95% limits of agreement = −0.25 to 0.26). RC correlated negatively with PA compliance.

**Conclusion:** This echo-Doppler method yields reasonable measurement of reflected wave in the pulmonary circulation, paving the way to a more integrative assessment of pulmonary hemodynamics in the clinical setting.

## Introduction

Pulmonary hypertension (PH) is a serious disease characterized by increased pulmonary artery pressure (PAP) associated with pulmonary vascular remodeling. It causes right-sided heart failure due to increased afterload on the right ventricle and has been shown to carry poor prognosis (Benza et al., 2012). Mean PAP and pulmonary vascular resistance (PVR) are the most common hemodynamic measures used to diagnose PH and monitor treatment effects. However, mean PAP used in isolation cannot characterize the severity of disease or define the pathological process, since it changes depending on various hemodynamic factors such as cardiac output (Hoeper et al., 2013), and as the disease progresses, an increase in PAP usually lags behind pathological changes in the pulmonary arterial tree, leading to a diagnostic delay (Lau et al., 2011). Mean PAP and PVR have been shown to correlate, but not closely, with the degree of right ventricular dysfunction and adverse clinical outcomes (Kawut et al., 2009; Maron et al., 2020). These inherent limitations may be because they do not represent all components of load faced by the right ventricle.

Pulmonary blood flows are pulsatile in nature. A complete description of right ventricular afterload, therefore, should also include the load to pulsatile component. In the pulmonary circulation, it accounts for approximately 25% of the total workload of right ventricle (Saouti et al., 2010); the proportion is much higher than that of the systemic circulation, because of lower vascular resistance in the pulmonary circulation. Once pathological changes develop in the pulmonary vasculature, they cause premature wave reflection from distal sites of pulmonary arterial trees, leading to an additional increase in pulsatile load (Hashimoto et al., 2008). Wave reflection has been studied in the systemic circulation, where altered wave reflection has been shown to be associated with the development of left ventricular hypertrophy and adverse clinical outcomes in left-sided heart failure (Hashimoto et al., 2008; Chirinos et al., 2012). Analysis of pulmonary arterial wave reflection could also provide additional information above and beyond that obtained from mean PAP and PVR, thereby helping better identify or treat patients with PH.

One promising method of estimating the magnitude of wave reflection is to separate pressure into its forward and backward components by wave separation analysis (Hollander et al., 2001). This analysis requires measurements of both pressure (P) and flow velocity (U) waveforms. Previous animal and clinical studies measured these two waveforms directly with catheters (Fukumitsu et al., 2017; Su et al., 2017; Yoshida et al., 2021). In clinical practice, pulmonary hemodynamics is assessed using Doppler echocardiography, for example, by estimating PAP from tricuspid regurgitation (TR) velocity and evaluate the pattern of flow profile at right ventricular outflow tract (RVOT) (Galie et al., 2015). Here we propose a new echo-Doppler method for assessing pulmonary artery wave reflection, providing a more detailed description of pulmonary hemodynamics than the present echocardiographic assessment. The specific objectives of this study were (1) to validate the echo-Doppler method against invasive wave separation analysis in a canine PH model induced by acute pulmonary micro-embolization, (2) to examine the influence of loading conditions on the derived indices, (3) to identify the hemodynamic determinants of the derived indices and (4) to explore the relationships between the derived indices and right ventricular function.

## Materials and Methods

### Principles of Echo-Doppler Method of Assessing Pulmonary Arterial Wave Reflection

Pulmonary arterial wave reflection can be assessed based on the concept of wave separation analysis. This analysis determines the origin, type and timing of traveling waves in a circulation from combined P and U measurements and allows wave separation into forward-traveling and backward-traveling components. Theoretical background and practical applications have been described elsewhere (Hollander et al., 2001; Yoshida et al., 2021). Briefly, wave speed (WS), which represents the elastic properties of the local artery, can be determined by P-U loop method (Khir et al., 2001); it takes advantage of the water hammer equation relating P and U on the condition that there is no wave reflection.


(1) $$\text{c}=\frac{1}{\rho }\sqrt{\frac{\sum {\text{dP}}^{2}}{\sum {\text{dU}}^{2}}},$$

where *d*P and *d*U are the changes in P and U, ρ is the density of blood (1050 kg/m^3^) and c is WS. Pulse pressure can be separated into those attributed to forward-traveling (Pf) and backward-traveling (Pb) waves using equations 2 and 3.


(2) $${\text{P}}_{\text{f}}=\left(P+\rho cU\right)/2$$

(3) $${\text{P}}_{\text{b}}=\left(P-\rho \mathrm{cU}\right)/2$$

The new noninvasive method we propose herein uses echo-Doppler derived P and U waveforms, instead of the direct measurements. A pulsed-wave Doppler tracing of RVOT flow is used as a surrogate for U waveform. On the other hand, P waveform is estimated by applying the simplified Bernoulli equation to an instantaneous Doppler velocities of TR jet and adding a term of right atrial pressure as below. This clinical equation has been derived from the more complex Bernoulli equation by assuming that viscous losses and acceleration effects are negligible and by using an approximation for the constant that relates to the mass density of blood, a conversion factor for measurement units (Baumgartner et al., 2009).


(4) $$\text{P}\left(\text{t}\right)=4TRV{\left(t\right)}^{2}+\mathrm{RAP},$$

where it is time, TRV is TR velocity and RAP is right atrial pressure which we assume is constant throughout the cardiac cycle.

End diastolic P is determined as *P*-value at the beginning of ejection (shown as t0) identified from the U waveform.


(5) $$\text{End}\mathrm{diastolic}P=4TRV{\left(\mathrm{t0}\right)}^{2}+\mathrm{RAP}$$

Subtracting end-diastolic P from P waveform yields pulse pressure waveform; this subtraction eliminates the term of right atrial pressure.


(6) $$\mathrm{Pulse}\mathrm{pressure}\left(\text{t}\right)=4\mathrm{TRV}{\left(t\right)}^{2}-4\mathrm{TRV}{\left(\mathrm{t0}\right)}^{2}$$

Wave separation analysis is performed on the pulse pressure and flow velocity waveforms by synchronizing waveforms of TR flow and RVOT flow using electrocardiogram. The pulse pressure waveform will then be separated into Pf and Pb. This analysis yields 1 arterial stiffness and 3 wave reflection indices: WS, peak Pb, peak Pf, and reflection coefficient (RC) calculated as the ratio of peak Pb to peak Pf.

### Animal Preparation

The present study was approved by the Animal Experimental Committee of Tokyo University of Agriculture and Technology (approval number: 30-146). All process of the study was carried out in compliance with the ARRIVE (Animal Research: Reporting of *in vivo* Experiments) guidelines and the regulations on animal experiments and the guide for the care and use of laboratory animals of Tokyo University of Agriculture and Technology (Percie du Sert et al., 2020). Eight healthy Beagle dogs (Kitayama Labes, Nagano, Japan) were used in the present study (all females, aged 4–5 years old, weighted 10–13 kg). All dogs were sedated with butorphanol (0.2 mg/kg), midazolam hydrochloride (0.2 mg/kg) and meloxicam (0.2 mg/kg) and initially anesthetized with propofol (4 mg/kg) before intubated and mechanically ventilated. Complete anesthesia was maintained by inhalation of isoflurane (end-tidal concentration 1.5 ± 0.1%). The dog was then placed in right lateral recumbency. Heparin sodium (100 IU/kg) was administered for preventing thrombosis.

A 4-Fr catheter (Atom nutrition catheter, Atom Medical, Tokyo, Japan) was inserted into the right femoral artery to monitor systemic arterial pressure. A 4.2-Fr multipurpose angiographic catheter (Goodtec angiographic catheter, GOODMAN, Aichi, Japan) was advanced retrograde into the left atrium through the left carotid artery to monitor left atrial pressure. Another 4.2-Fr multipurpose angiographic catheter was placed in the right atrium through the left jugular vein to monitor right atrial pressure. These fluid-filled catheters were connected to pressure transducers (Life kit DX-360, Nihon Kohden, Tokyo, Japan) and pressure waveforms were displayed using a multi-channel monitor (Life Scope BSM-5192; Nihon Kohden, Tokyo, Japan). These two catheters were also used to sample blood from the left and right atriums to calculate cardiac output.

To obtain P and U waveforms as a reference for validating the new echo-Doppler method, a dual sensor-tipped pressure and flow wire (Combowire, Royal Philips, Amsterdam, Netherlands) was advanced to approximately 1 cm beyond the pulmonary valve through another 4.2-Fr multipurpose angiographic catheter inserted from the left jugular vein. Careful catheter and wire manipulation ensured that signals were steadily obtained.

### Experimental Protocol

**FIGURE 1:**
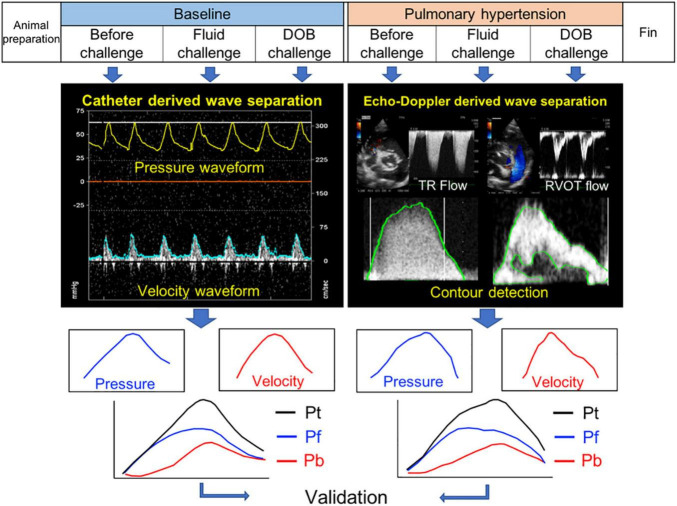
Study design. Pt, Pf, and Pb denote pulse pressure, forward pressure and backward pressure, respectively.

The study design is outlined in Figure 1. To assess whether the new echo-Doppler method can detect the alterations in pulmonary arterial wave reflection associated with the development of PH, each dog was given repeated injections of dextran microsphere cross-linked with epichlorohydrine (Sephadex G-50, GE healthcare, diameter 300 μm) until mean PAP increased above 30 mmHg. To investigate the influences of hemodynamic alterations on wave reflection, each dog received separate continuous drip infusions of lactated Ringer’s solution until mean right atrial pressure increased by 2 mmHg (fluid loading) and dobutamine (inotropic stimulation, 3 μg/min/kg), both at baseline and after the induction of PH. Every time a single intervention of either fluid or dobutamine challenge was finished, we waited until pulmonary arterial hemodynamics became back to the baseline before moving onto the next state-load condition. As a result, each stage took at least 30 min. At each state-load condition, the direct P and U measurements and echocardiographic image acquisitions as well as pulmonary hemodynamic assessment were conducted, as described later in this section.

### Hemodynamic Assessment and Direct Pressure and Flow Measurement

Cardiac output was calculated by Fick method from oxygen content of blood sampled from the left and right atriums and oxygen consumption estimated using Sykes’ formula (Percie du Sert et al., 2020). Stroke volume was calculated as cardiac output / heart rate. PVR was calculated as (mean PAP - mean left atrial pressure) / cardiac output. Pulmonary artery compliance (PAC) was approximated as stroke volume / (systolic PAP – diastolic PAP).

P and U data were acquired simultaneously for 30 to 60 s during end-expiration apnea, using the dual sensor-tipped catheter. The sampling rate at the acquisition was 200 Hz (5-msec temporal resolution). The data were then exported digitally for subsequent wave separation analysis.

### Echocardiographic Image Acquisition

Comprehensive echocardiographic examination was performed, using a ProSound F75 premier CV with a 5-MHz transducer (Hitachi, Tokyo, Japan). Left ventricular ejection fraction were measured by the disk summation method. To assess right ventricular systolic function, TAPSE was measured in an apical 4-chamber view. A pulsed-wave Doppler tracing of RVOT flow and a continuous-wave Doppler tracing of TR jet were recorded at a sweep speed of 300 mm/s to assess pulmonary hemodynamics. Careful attention was paid to obtain clear spectral Doppler envelopes. TR jet was able to be detected in all cases, because angiographic catheters passed across the tricuspid valve may create TR. To assess the severity of PH, peak TR velocity was measured. The time from the onset of ejection to peak TR velocity was also measured to evaluate peak pattern of TR velocity profile. From the RVOT flow profile, acceleration time was measured as the time from the onset of ejection to the peak velocity. Mid-systolic notching was considered present, if there was a distinct notch in the mid portion of the RVOT flow profile, dividing the flow profile into two different peaks. These Doppler echocardiographic images were stored in a digital format for subsequent echo-Doppler based wave separation analysis.

### Wave Separation Analysis

The above echocardiographic images were processed using in-house program code written in MATLAB (MathWorks 2019b, MA, United States). The envelope of RVOT flow Doppler signal was traced semi-automatically to obtain U waveform. This waveform was smoothed using a Savitzky–Golay filter and then ensemble averaged over 3 cardiac cycles with reference to the R wave on electrocardiogram. The timing of beginning and end of ejection were identified from the U waveform. P waveform was estimated by applying the simplified Bernoulli equation to a semi-automatic tracing of the envelope of TR Doppler signal. This waveform was smoothed using a Savitzky–Golay filter and then ensemble averaged over 3 cardiac cycles with reference to the R wave on electrocardiogram. Pulse pressure waveform was obtained by subtracting end-diastolic P from the P waveform [equation (8)]. The temporal resolution for both waveforms was approximately 2.0 msec for all acquisitions that resulted from the setting of sweep speed at the acquisition (300 mm/s) and a pixel size of the echocardiographic images. The velocity resolution was dependent on the Nyquist limit and a pixel size of the echocardiographic images; this yielded a velocity resolution of 0.5 ± 0.7 cm/s for RVOT flow and 1.4 ± 0.4 cm/s for TR jet. The pressure resolution varied depending on the level of pressure, since pressure was estimated proportional to the square of velocity. The waveforms were then interpolated using cubic spline and resampled to 1 msec temporal resolution (1,000 Hz).

The reference P and U waveforms measured directly with the catheter were processed similarly as were the echo-Doppler waveforms. The waveforms were smoothed using a Savitzky–Golay filter and then ensemble averaged over 3 cardiac cycles with reference to the R wave on electrocardiogram. These waveforms were then interpolated using cubic spline and resampled to 1 msec temporal resolution (1,000 Hz). The timings of the beginning and end of ejection were identified from the U waveform. End-diastolic P was subtracted from the P waveform to obtain pulse pressure waveform before wave separation analysis.

Wave separation analysis was performed separately on the echo-Doppler derived waveforms and reference waveforms, using in-house program code written in MATLAB. To determine WS, pulse pressure was plotted against velocity over the ejection period. At the beginning of ejection, a linear portion was always observed on the P-U loop, suggesting that a wavefront during this period was only that generated by the contraction of right ventricle. The slope of the linear portion was calculated from the loop and divided by the density of blood to obtain WS. Pulse pressure waveform was then separated into Pf and Pb waveforms, as described earlier in this section. In this study, Pf and Pb were presented as the peak value and these peak values were used for calculating RC.

### Statistical Analysis

Categorical variables were expressed as number and percentage and were compared using Fisher’s exact test. Continuous variables were expressed as mean ± standard deviation. After an assessment of the differences in the before challenge, fluid challenge, and dobutamine challenge results using two-way repeated-measures ANOVA, Tukey’s honestly significant difference *post hoc* test was used to compare variables between each result. Linear regression analysis was used to compare echo-Doppler derived wave reflection and arterial stiffness indices against catheter derived indices. Bland-Altman plot was used to visualize random and systematic errors. Linear regression analyses were also performed to identify the hemodynamic determinants of wave reflection and arterial stiffness indices. Explanatory variables included PVR, pulmonary arterial compliance, cardiac output and heart rate. Analysis of covariance was used to determine the effect of fluid loading and inotropic stimulation on the relationship between wave reflection and arterial stiffness indices and right ventricular systolic function. *P-*value < 0.05 was considered as statistically significant. All statistical analyses were performed using IBM SPSS statistics version 19 (IBM, IL, United States) and GraphPad Prism version 8.0 (GraphPad, California, CA, United States).

## Results

### Pulmonary Arterial Hemodynamics and Wave Reflection

The changes in pulmonary hemodynamics and right ventricular function induced by pulmonary arterial microembolization and hemodynamic manipulations are summarized in Table 1. The injection of dextran microsphere significantly increased mean PAP from 14 ± 3 to 34 ± 7 mmHg (*p* = 0.008) and PVR from 2.9 ± 1.0 to 13.7 ± 4.9 Woods unit (*p* = 0.011). Cardiac output decreased (*p* = 0.04). Right ventricular systolic function was also reduced, as demonstrated by a significant decrease in tricuspid annular plane systolic excursion (TAPSE) (*p* = 0.015). There were alterations in RVOT flow profile such as the presence of mid-systolic notching; TR jet profile exhibited high and late peaking pattern as shown by higher TR velocity (*p* = 0.006) and longer time to peak velocity (*p* = 0.01). Fluid loading did not change PVR (at baseline, *p* = 0.99; at PH state, *p* = 0.63); it did not increase cardiac output at both baseline (*p* = 0.46) or not at all at PH state (*p* = 0.61). Inotropic stimulation did not change PVR (at baseline, *p* = 0.93; at PH state, *p* > 0.99), although it significantly increased cardiac output at baseline (*p* < 0.001), but did not increase cardiac output at PH state (*p* = 0.36).

**TABLE 1:** The changes in hemodynamic variables obtained invasively and echocardiographic variables, wave reflection variables.

	Baseline			Pulmonary hypertension		
	Before challenge	Fluid challenge	DOB challenge	Before challenge	Fluid challenge	DOB challenge
**Hemodynamic variables**						
SAP, mmHg						
Systolic	110 ± 9	108 ± 17	111 ± 16	97 ± 16*	96 ± 14	106 ± 15
Diastolic	89 ± 16	83 ± 23	81 ± 26	76 ± 18*	73 ± 19	84 ± 18
Mean	93 ± 17	88 ± 17	95 ± 20	83 ± 18*	83 ± 19	95 ± 17
LAP, mmHg	6 ± 1	7 ± 1	7 ± 1	6 ± 2	8 ± 3	6 ± 2
**PAP, mmHg**						
Systolic	21 ± 4	25 ± 6	28 ± 12	47 ± 7*	49 ± 14	63 ± 22
Diastolic	11 ± 3	13 ± 5	15 ± 10	28 ± 8*	28 ± 16	31 ± 14
Mean	14 ± 3	17 ± 5	19 ± 11	34 ± 7*	35 ± 15	42 ± 16
RAP, mmHg	4 ± 2	6 ± 2*	4 ± 3	5 ± 2	7 ± 2^†^	5 ± 3
PA flow, cm/s	72 ± 9	85 ± 11	88 ± 22	73 ± 15	70 ± 11	76 ± 15
HR, /min	126 ± 7	130 ± 21	126 ± 22	124 ± 12	131 ± 17	137 ± 12^†^
CO, L/min	2.7 ± 0.1	2.9 ± 0.3	3.2 ± 0.1*	2.1 ± 0.5*	2.2 ± 0.4	2.4 ± 0.5
SV, ml	22 ± 1	23 ± 5	26 ± 4*	17 ± 4*	17 ± 4	18 ± 4
PVR, Wood units	2.9 ± 1.0	3.2 ± 2.1	3.5 ± 2.1	13.7 ± 4.9*	12.1 ± 5.2	14.6 ± 5.4
PAC, ml/mmHg	2.1 ± 0.5	2.0 ± 0.6	2.0 ± 0.3	1.1 ± 0.6*	0.9 ± 0.4	0.6 ± 0.2
**Echocardiographic variables**						
LVEF, %	66.0 ± 6.5	77.8 ± 7.7	81.7 ± 5.9	74.5 ± 9.8	80.4 ± 8.6	79.8 ± 19.9
TAPSE, mm	11.2 ± 2.1	12.1 ± 2.3	13.0 ± 1.0	9.3 ± 2.1*	9.6 ± 1.5	10.1 ± 1.3
**RVOT flow**						
Peak velocity, cm/s	76 ± 14	87 ± 22	96 ± 25	75 ± 11	77 ± 15	89 ± 20
AcT, ms	93 ± 23	99 ± 20	88 ± 18	71 ± 14	71 ± 14	73 ± 11
Mid-systolic notching, %	0	0	0	50	63	63
**TR jet**						
Peak velocity, m/s	2.0 ± 0.2	2.2 ± 0.4	2.4 ± 0.4	3.1 ± 0.4*	3.4 ± 0.5	3.9 ± 0.6^†^
Time to peak, ms	135 ± 32	132 ± 33	125 ± 41	185 ± 35*	182 ± 51	185 ± 56
Estimation of PAP, mmHg	21 ± 4	24 ± 5	28 ± 8	43 ± 16*	51 ± 16	66 ± 17
Wave reflection variables						
Catheter						
Pb, mmHg	1.9 ± 0.8	2.3 ± 0.9	2.3 ± 0.8	8.2 ± 2.6*	8.8 ± 2.7	12.4 ± 2.8^†^
Pf, mmHg	8.6 ± 1.5	9.9 ± 2.0	10.5 ± 2.4	14.6 ± 3.5	15.5 ± 3.0	22.6 ± 8.1
RC	0.21 ± 0.08	0.22 ± 0.10	0.23 ± 0.1	0.57 ± 0.12*	0.57 ± 0.13	0.58 ± 0.13
Wave speed, m/s	1.1 ± 0.2	1.2 ± 0.3	1.5 ± 0.3	2.6 ± 0.5*	3.0 ± 0.5	3.2 ± 0.4
Echocardiography						
Pb, mmHg	1.4 ± 1.3	1.8 ± 0.7	3.0 ± 1.3	10.4 ± 3.9*	9.8 ± 4.7	11.9 ± 3.0
Pf, mmHg	8.6 ± 2.5	9.8 ± 2.5	10.1 ± 1.6	16.1 ± 5.1	16.3 ± 3.9	22.5 ± 5.3^†^
RC	0.16 ± 0.1	0.18 ± 0.05	0.29 ± 0.11	0.65 ± 0.13*	0.58 ± 0.14	0.55 ± 0.14
Wave speed, m/s	1.2 ± 0.3	1.5 ± 0.6	1.5 ± 0.4	3.3 ± 1.0*	3.4 ± 1.1	3.6 ± 1.0

SAP, systemic arterial pressure; LAP, left atrial pressure; PAP, pulmonary arterial pressure; RAP, right atrial pressure; HR, heart rate; CO, cardiac output; SV, stroke volume; PVR, pulmonary vascular resistance; PAC, pulmonary arterial compliance; LVEF, left ventricular ejection fraction; TAPSE, tricuspid annular plane systolic excursion; RVOT, right ventricular outflow tract; AcT, acceleration time; TR, tricuspid regurgitation; Pb, backward pressure; Pf, forward pressure; RC, reflection coefficient; WS, wave speed; * denotes p-value < 0.05 vs. Baseline before challenge and ^†^ denotes p-value < 0.05 vs. Pulmonary hypertension before challenge.

### Correlations of Wave Reflection and Arterial Stiffness Indices by Doppler With Those by Catheter

**FIGURE 2:**
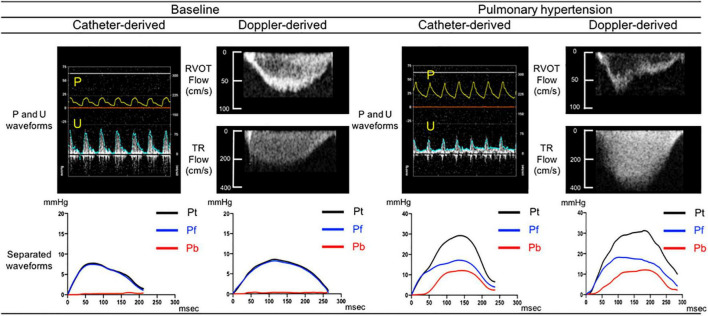
Comparison between catheter derived and Doppler derived separated pressure waveforms. Separated pressure waveforms obtained using direct pressure and flow measurements acquired from one sample dog (left side) and those obtained using echo-Doppler images of RVOT and TR flows from the same dog (right side). Pt, Pf, and Pb denote pulse pressure, forward pressure and backward pressure, respectively.

Figure 2 compares sample separated waveforms obtained from direct P and U measurements and those obtained from echo-Doppler derived P and U measurements. At baseline, almost no wave reflection was observed. After the induction of PH, pulse pressure by Doppler dramatically increased from 7.7 to 29.2 mmHg with late-peaking pattern; Pb by Doppler started to rise 52 msec later than the onset of ejection and peaked at 12.1 mmHg. This comparison demonstrated that the new echo-Doppler method produced comparable Pb and Pf waveforms with those derived from direct measurements. Overall, Pb by Doppler was not zero, but minimal (1.4 ± 1.3 mmHg) at baseline and increased 7.5 times, up to 10.4 ± 3.9 mmHg, associated with the development of PH, even though mean PAP increased only 2.4 times, up to 34 ± 7 mmHg; its increase was substantially larger, compared to 1.9 times increase in Pf by Doppler (from 8.6 ± 2.5 to 16.1 ± 5.1 mmHg), resulting in a significant rise in RC by Doppler (from 0.16 ± 0.10 to 0.65 ± 0.13). WS by Doppler also significantly increased from 1.2 ± 0.3 to 3.3 ± 1.0 m/s.

**FIGURE 3:**
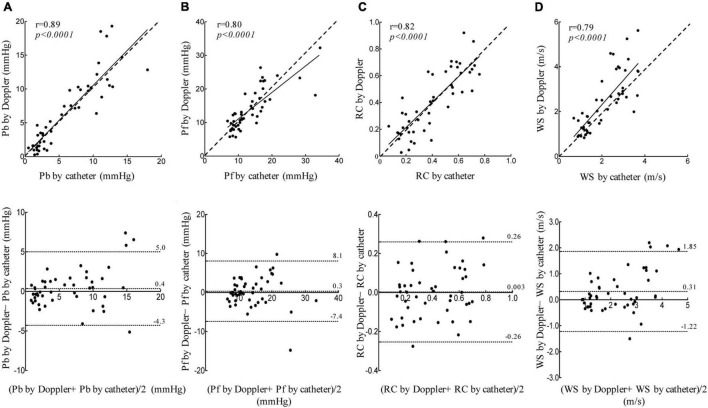
Linear regression and Bland-Altman analyses. Linear regression (top figure, dashed line represents identical line.) and Bland-Altman (bottom figure, middle dashed line represents mean difference; dashed line of upper and lower represents the limits of agreement.) plots comparing catheter derived and echo-Doppler derived Pb **(A)**, Pf **(B)**, RC **(C)**, and WS **(D)**. The limits of agreement are defined as the mean difference ± 1.96 SD of differences.

**FIGURE 4:**
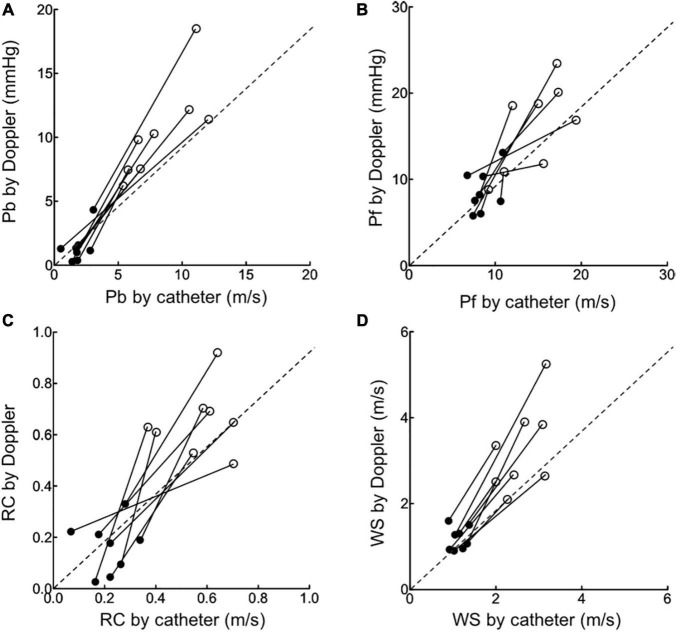
The changes in wave reflection and arterial stiffness indices induced by pulmonary hypertension. The changes in echo-Doppler derived Pb **(A)**, Pf **(B)**, RC **(C)** and WS **(D)** from baseline (black circle) to pulmonary hypertension state (open circle) were compared to those in corresponding catheter derived indices.

When the data for all load-state conditions were pooled, Pb, Pf, and RC by Doppler correlated linearly with the corresponding indices by catheter with acceptable variabilities (Figure 3). Lower part of Figure 3 also shows the result of a Bland-Altman analysis between the Pf (Mean difference, 0.3; SD of mean difference, 3.94; the limits of agreement, −7.4 to 8.1), Pb (Mean difference, 0.4; SD of mean difference, 2.36; the limits of agreement, −4.3 to 5.0), RC (Mean difference, 0.003; SD of mean difference, 0.13; the limits of agreement, −0.26 to 0.26), WS (Mean difference, 0.31; SD of mean difference, 0.78; the limits of agreement, −1.22 to 1.85) evaluated by the catheter and Doppler methods. Almost of all dot plots did not exceed the limits of agreement. WS by Doppler also significantly correlated with that by catheter; the regression line in the scatterplot was deviated slightly upward from the identical line, suggesting that the echo-Doppler method overestimated WS. When looking at individual change in wave reflection and arterial stiffness indices for each dog, the induction of PH increased the indices by Doppler similarly as did those by catheter (Figure 4). This indicated that the echo-Doppler method was able to track the alterations in pulmonary arterial wave reflection associated with the development of PH, with reasonable accuracy. On the other hand, Bland-Altman analysis also showed that the error tended to increase as the wave reflection increased.

### Wave Reflection and Arterial Stiffness Indices and Effects of Hemodynamic Alterations

Table 2 summarizes the changes in wave reflection and arterial stiffness indices by Doppler induced by preload loading and inotropic stimulation. An increase in preload by fluid loading, which caused a significant, but small increase in cardiac output (Table 1, Baseline Fluid challenge), did not alter the wave reflection and arterial stiffness indices. In Table 2, the result of fluid challenge is following: Pb in Fluid challenge Baseline before vs. after, *p* = 0.4; Pb in Fluid challenge Pulmonary hypertension before vs. after, *p* = 0.57; Pf in Fluid challenge Baseline before vs. after, *p* = 0.4; Pf in Fluid challenge Pulmonary hypertension before vs. after, *p* = 0.89; RC in Fluid challenge Baseline before vs. after, *p* = 0.44; RC in Fluid challenge Pulmonary hypertension before vs. after, *p* = 0.16; WS in Fluid challenge Baseline before vs. after, *p* = 0.23; WS in Fluid challenge Pulmonary hypertension before vs. after, *p* = 0.84. Inotropic stimulation produced a larger increase in cardiac output than fluid loading (Table 1, Baseline DOB challenge); it increased Pb as well as Pf, but did not alter RC. In table 2, the result of dobutamine challenge was following: Pb in dobutamine challenge baseline before vs. after, *p* = 0.08; Pb in dobutamine challenge pulmonary hypertension before vs. after, *p* = 0.14; Pf in dobutamine challenge baseline before vs. after, *p* = 0.17; Pf in dobutamine challenge pulmonary hypertension before vs. after, *p* = 0.03; RC in dobutamine challenge baseline before vs. after, *p* = 0.11; RC in dobutamine challenge pulmonary hypertension before vs. after, *p* = 0.08; WS in dobutamine challenge baseline before vs after, *p* = 0.23; WS in dobutamine challenge pulmonary hypertension before vs. after, *p* = 0.34. These results suggested that both Pb and Pf were flow-dependent and the effect of flow status on Pb can be cancelled out by taking a ratio of it to Pf. Inotropic stimulation also did not alter WS. These trends were also confirmed for catheter-derived wave reflection and arterial stiffness indices (Supplementary Table 1). In Supplementary Table 1, the result of Fluid challenge was following: Pb in Fluid challenge Baseline before vs. after, *p* = 0.18; Pb in Fluid challenge Pulmonary hypertension before vs. after, *p* = 0.54; Pf in Fluid challenge Baseline before vs. after, *p* = 0.11; Pf in Fluid challenge Pulmonary hypertension before vs. after, *p* = 0.51; RC in Fluid challenge Baseline before vs. after, *p* = 0.53; RC in Fluid challenge Pulmonary hypertension before vs. after, *p* = 0.8; WS in Fluid challenge Baseline before vs. after, *p* = 0.3; WS in Fluid challenge Pulmonary hypertension before vs. after, *p* = 0.11 (Inotropic stimulation increased Pb and Pf, but did not change RC). In Supplementary Table 1, the result of dobutamine challenge was following: Pb in Dobutamine challenge Baseline before vs. after, *p* = 0.03; Pb in Dobutamine challenge Pulmonary hypertension before vs. after, *p* = 0.01; Pf in Dobutamine challenge Baseline before vs. after, *p* = 0.03; Pf in Dobutamine challenge Pulmonary hypertension before vs. after, *p* = 0.04; RC in Dobutamine challenge Baseline before vs. after, *p* = 0.62; RC in Dobutamine challenge Pulmonary hypertension before vs. after, *p* = 0.78; WS in Dobutamine challenge Baseline before vs. after, *p* = 0.07; WS in Dobutamine challenge Pulmonary hypertension before vs. after, *p* = 0.07.

**TABLE 2:** Effects of hemodynamic manipulations on Doppler-derived.

Fluid challenge				
	Baseline		Pulmonary hypertension	
	Before	After	Before	After
Pb, mmHg	1.4 ± 1.3	1.8 ± 0.7	10.4 ± 3.9	9.8 ± 4.7
Pf, mmHg	8.6 ± 2.5	9.8 ± 2.5	16.1 ± 5.1	16.3 ± 3.9
RC	0.16 ± 0.10	0.18 ± 0.05	0.65 ± 0.13	0.58 ± 0.14
WS, m/s	1.2 ± 0.3	1.5 ± 0.6	3.3 ± 1.0	3.4 ± 1.1

**Dobutamine challenge**				

	**Baseline**		**Pulmonary hypertension**	
	**Before**	**After**	**Before**	**After**

Pb, mmHg	1.5 ± 1.6	3.0 ± 1.3	11.1 ± 2.2	11.9 ± 3.0
Pf, mmHg	9.1 ± 1.9	10.1 ± 1.6	15.1 ± 4.4	22.5 ± 5.3*
RC	0.15 ± 0.22	0.29 ± 0.11	0.61 ± 0.22	0.55 ± 0.14
WS, m/s	1.4 ± 0.5	1.5 ± 0.4	3.4 ± 1.3	3.6 ± 1.0

Pb, backward pressure; Pf, forward pressure; RC, reflection coefficient; WS, wave speed; * denotes p-value < 0.05 vs. before challenge.

### Hemodynamic Determinants of Wave Reflection and Arterial Stiffness Indices

The results of linear regression analyses identifying the determinants of echo-Doppler derived wave reflection and arterial stiffness indices are shown in Table 3. RC was correlated strongly with PAC, moderately with PVR, and weakly with cardiac output. WS was also correlated strongly with PAC and PVR, moderately with cardiac output. The magnitude of the associations with both RC and WS was the highest for PAC (RC by Doppler, *r* = −0.85; WS by Doppler, *r* = −0.71). Stepwise multiple linear regression analysis revealed that PAC and cardiac output were independent determinants of RC, and PAC was an independent determinant of WS. PAC was also identified as the strongest hemodynamic determinant for catheter-derived RC and WS (RC, *r* = −0.70; WS, *r* = −0.79, Supplementary Table 2).

**TABLE 3:** Hemodynamic determinants of Doppler-derived wave reflection indices.

	RC		WS	
	*R*	*P*-value	*R*	*P*-value
PVR	0.58	< 0.001	0.62	< 0.001
PAC	–0.85	< 0.001	–0.71	< 0.001
CO	–0.34	0.02	–0.59	< 0.001
HR	0.26	0.08	0.06	0.68
sPAP	0.74	< 0.001	0.58	< 0.001
mPAP	0.55	< 0.001	0.48	0.001
mLAP	–0.17	0.250	–0.09	0.53

*RC, reflection coefficient; WS, wave speed; PVR, pulmonary vascular resistance; PAC, pulmonary arterial compliance; CO, cardiac output; SV, stroke volume; HR, heart rate; sPAP, systolic pulmonary arterial pressure; dPAP, diastolic pulmonary arterial pressure; mPAP, mean pulmonary arterial pressure; mLAP, mean left atrial pressure.*

### Right Ventricular - Arterial Coupling by Wave Reflection and Arterial Stiffness Indices

**FIGURE 5:**
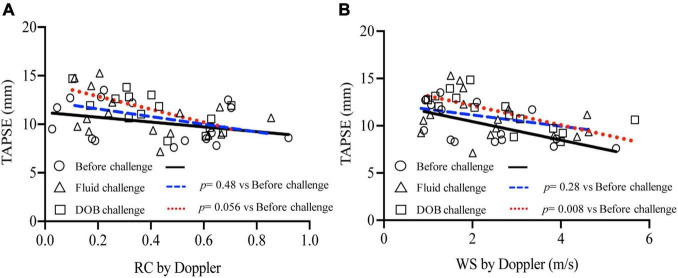
Relationship between wave reflection and arterial stiffness indices and RV systolic function. TAPSE was plotted against echo-Doppler derived RC **(A)** and WS **(B)**, with separate regression lines for data acquired before challenge (solid line), during fluid challenge (dashed line) and during dobutamine challenge (dotted line).

Figure 5 relates right ventricular systolic function measured by TAPSE with echo-Doppler derived wave reflection and arterial stiffness indices. Overall, TAPSE was negatively correlated with both RC (*r* = −0.40, *p* = 0.005) and WS (*r* = −0.49, *p* = 0.001). When analyzed separately for loading conditions, the relationships of TAPSE with RC and WS did not change by fluid loading (RC, *p* = 0.48; WS, *p* = 0.28). However, the relationships were shifted upward by inotropic stimulation (RC, *p* = 0.06; WS, *p* = 0.008). When catheter-derived RC and WS were used instead for the above analysis, similar upward shifts by inotropic stimulation were observed (Supplementary Figure 1).

## Discussion

The present study investigated that pulmonary arterial wave reflection can be assessed noninvasively using Doppler echocardiography. Pulmonary arterial wave reflection was gained by performing wave separation analysis to separate pulmonary arterial pressure waveform estimated from TR velocity profile into its forward and backward components. As the result, we reported that the echo-Doppler method yields reasonable measurement of pulmonary arterial wave reflection and may detect the alterations associated with the development of PH.

### Noninvasive Assessment of Pulmonary Arterial Wave Reflection

To infer a diagnosis of PH, the effects of PH on the right heart are imaged with echocardiography in clinical practice. Some of such echocardiographic signs are related to the presence of pulmonary arterial wave reflection. For example, a late-peaking pattern on TR velocity profile is caused by PAP augmentation by early arrival of reflected wave (Bech-Hanssen et al., 2010). A mid-systolic notching on RVOT flow profile represents an abrupt reduction in right ventricular ejection flow secondary to wave reflection (López-Candales and Edelman, 2012). Although these signs are reasonably specific, the diagnostic sensitivity is generally low and these signs are absent in some patients (Arkles et al., 2011). The signs are also not sensitive enough to be used for monitoring the treatment course of patients.

To provide a more accurate assessment of wave reflection, wave separation analysis is performed on pulmonary arterial pressure and flow waveforms into their forward and backward components. This analysis is classically undertaken in the frequency domain, but it is fairly complicated and needs sophisticated software (Segers et al., 2007). An alternative method, called wave separation analysis, has been particularly attracting attention, because it is a time-domain based approach and therefore can be more easily applied to study the timing and magnitude of wave reflection (Laxminarayan, 1979; Li, 1986). The results are easier to interpret in physiological terms.

Most previous studies of pulmonary arterial wave separation analysis measured pressure and flow waveforms directly with catheters, which hinders its use in the clinical setting (Hollander et al., 2001). A recent study has attempted to obtain these waveforms noninvasively, using magnetic resonance imaging, where It measured the temporal changes in cross-sectional area of pulmonary artery for a surrogate for PAP (Quail et al., 2015). The noninvasive method we introduce herein uses Doppler echocardiography to obtain pulmonary arterial pressure and flow waveforms from TR and RVOT flow profiles. These measurements are routinely performed for diagnosing PH or monitoring the treatment course, so this echo-Doppler method can be easily applied in the clinical setting.

The magnitude of wave reflection was usually quantified by RC that was defined as a ratio of backward to forward component of either energy of waves or pressure. This study has reported that RC was not zero, but small at 0.16 ± 0.10 at baseline in dogs. This may be because dogs have inherently a mild degree of PH with a PVR of 2.9 ± 1.0 Woods unit. This finding suggested that wave separation analysis may be sensitive for detecting a mild degree of PH. This study also found that RC correlated negatively with right ventricular systolic function. This negative correlation suggested that RC was able to reasonably capture right ventricular – arterial coupling in the acute setting. The experimental model in this study was different from typical clinical scenarios, i.e., a long-term adaptation or maladaptation of pulmonary vasculature and right ventricular myocardium in response to chronic pressure loading. Thus, it needs to be tested whether RC was able to describe the coupling in the clinical setting.

This echo-Doppler method involves several assumptions. Firstly, right atrial pressure is assumed to be constant during systole. The fluctuation in right atrial pressure during this period is usually small enough to be negligible, when compared with PAP. This assumption seemed valid from the results of this validation study demonstrating that the echo-Doppler method accurately quantified wave reflection. It may be because all dogs used in this study presented with only mild degree of TR. Once severe, it causes a significant rise in right atrial pressure in late systole; thus, we assume that the echo-Doppler method may not be applicable to those with severe TR. Secondly, a pulsed-wave Doppler tracing of RVOT flow was used as a surrogate for PA flow velocity waveform. The velocity should be higher when measured at RVOT than when measured at pulmonary arterial trunk, which should result in higher characteristic impedance, i.e., wave speed when assessed by the echo-Doppler method. This overestimation was confirmed by the present study showing that wave speed measured by the echo-Doppler method was higher, by a mean of 0.42 m/s, than that by direct measurement. Thirdly, RR interval on electrocardiogram is assumed to be constant throughout image acquisitions. In this method, pressure and flow waveforms are obtained from echo-Doppler images acquired at different cardiac cycle and the both waveforms are aligned in time with reference to the R wave on electrocardiogram. Therefore, this method cannot be applied to patients with large heart rate variability such as atrial fibrillation. Fourthly, Hollander et al. reported the existence of negative reflections which occurs in early systole in dogs, indicating which casts doubt on the validity of early systole being reflection-free which is a necessary assumption for use of the Water Hammer equation. However, we think that the impact of this negative reflections in systole are small.

Despite the above assumptions, the present study demonstrated that the echo-Doppler method can quantify pulmonary arterial wave reflection with reasonable accuracy and it is sensitive for detecting the changes in pulmonary arterial wave reflection associated with the development of PH.

### Potential for Clinical Applications

Pulmonary arterial hypertension remains a fatal disease with a 1-year mortality of approximately 20%, despite advances in disease-specific therapies (Benza et al., 2012). This may be because the disease is often far established at diagnosis as demonstrated by contemporary disease registries reporting that mean PVR at diagnosis ranged between 8 and 10 Wood units (Thenappan et al., 2007). Early initiation of the therapies may be effective, as suggested by a previous randomized control study including patients with mildly symptomatic pulmonary arterial hypertension (Galiè et al., 2008). At the early stage of disease, PAC falls considerably while PVR increases only slightly. Thus, the assessment of pulsatile load may allow an early diagnosis even when PVR stays within normal limits. In the present study, wave reflection, an important component of the pulsatile load, rose substantially despite a slight increase in mean PAP induced by pulmonary arterial microembolization. This finding is consistent with another study of wave separation analysis, evidencing that a significant wave reflection was present even in PH patients with mildly elevated PAP (Su et al., 2017). Therefore, this echo-Doppler assessment of wave reflection may be able to offer an early diagnosis of PH.

PH-specific treatments do not always produce desirable clinical outcomes. For example, some patients with chronic thromboembolic pulmonary hypertension exhibit exercise intolerance even after pulmonary endarterectomy, often despite normalization of pulmonary arterial hemodynamics. This may partly be attributable to persistent pulmonary arterial wave reflection after the surgery. A previous study demonstrated using wave separation analysis that there remained a large reflected pressure wave 3 months after the surgery, even in patients without residual PH (Su et al., 2019). Pulmonary arterial wave reflection could be an attractive target for the treatment of PH (Su et al., 2018). However, it remained to be tested whether a reduction in pulmonary arterial wave reflection will lead to a significant improvement in clinical outcomes.

Wave reflection is one component of pulsatile load, so it is inherently included in arterial compliance. This is why RC correlated with pulmonary arterial compliance. Pulse pressure, a major component of arterial compliance, is a sum of forward pressure representing stiffness of proximal artery and backward pressure representing pathology of more peripheral arteries. When pulse pressure is elevated, we are not sure which of the two pressures is elevated. Therefore, assessing wave reflection may offer benefits over pulmonary arterial compliance for discriminating diseases with different sites of arteries affected, for example, primary pulmonary arterial hypertension and chronic thromboembolic pulmonary hypertension. While the clinical implication of wave reflection in the systemic circulation is becoming more apparent, only a few studies have applied wave reflection in the pulmonary circulation and these have mainly been in animal models (Hollander et al., 2001; Nie et al., 2001). Therefore, since the current concept of wave intensity analysis in the pulmonary circulation has been established by animal studies, the results of this study may be applicable in human clinical practice. The present study demonstrated that a noninvasive measurement of PA wave reflection is feasible using Doppler echocardiography. The methodology developed in our study may be potentially helpful in future clinical applications involving pathological changes in the distensibility of the pulmonary artery. Recent advances in imaging technologies should facilitate future use of pulmonary wave intensity analysis in a clinical setting. We expect PA wave reflection provides additional information about hemodynamic of pulmonary circulation.

## Limitations

The scale of verification in this study was small and that limits being able to affirm that the technique has been validated completely. In addition, to validate the echo-Doppler method, the present study created an animal model of PH which mimicked pulmonary arterial hypertension by embolizing peripheral pulmonary arterial with microsphere. The magnitude and timing of wave reflection vary depending on the site of narrowing in pulmonary vasculature; this was evidenced by previous studies of wave separation analysis observing a larger reflected wave that arrived earlier during systole in chronic thromboembolic pulmonary hypertension than that in pulmonary arterial hypertension (Castelain et al., 2001). It needs to be investigated whether or not the echo-Doppler method can detect the alterations in the magnitude and timing of pulmonary arterial wave reflection by changes in the site of narrowing. In addition, previous studies have analyzed arterial viscoelasticity and pulse wave propagation properties during acute pulmonary artery hypertension, indicating that the role of smooth muscle activation should be considered, as a key determinant of pulmonary artery wave reflection and right ventricular afterload (Bia et al., 2004; Grignola et al., 2007). Thus, in pulmonary vascular, activation of vascular smooth muscle must be considered as factor of changing wave reflection.

Brand-Altman analysis showed that the error tended to increase as the wave reflection increased. The estimates of the limits of agreement from Bland Altman analysis are likely to be underestimated due to the non-independence of data from the same animals.

Although TR was effectively induced by the catheter passing the tricuspid valve in this study, TR is not always evident in the clinical setting. Therefore, in clinical cases, measuring the PA wave reflection in cases that do not exhibit TR can be difficult. In addition, some studies questioned the accuracy of echocardiographic estimates of pulmonary arterial systolic pressure in the clinical setting, because TR velocity is sometimes difficult to measure or to obtain clear spectral Doppler envelopes (Fisher et al., 2009). Consequently, this echo-Doppler method should also be tested for feasibility and accuracy in patients before clinical application.

## Conclusion

The new echo-Doppler method yields reasonable measurement of pulmonary arterial wave reflection and can detect the alterations associated with the development of PH. This work shows that the methodological approach used could be useful for the non-invasive assessment of pulmonary wave reflections; although more animal research and subsequent research in humans are necessary

## Data Availability Statement

The original contributions presented in the study are included in the article/Supplementary Material, and further inquiries can be directed to the corresponding author/s.

## Ethics Statement

The animal study was reviewed and approved by Animal Experimental Committee of Tokyo University of Agriculture and Technology.

## Author Contributions

TYo and TYa designed the study and wrote the initial draft of the manuscript. SK and KM acquired and analyzed the data and performed the experiment. AU, HH, and ZY interpreted the results and critically revised the results. TU and RT edited the manuscript and approved the final version of the manuscript. All authors contributed to the article and approved the submitted version.

## Conflict of Interest

The authors declare that the research was conducted in the absence of any commercial or financial relationships that could be construed as a potential conflict of interest.

## Publisher’s Note

All claims expressed in this article are solely those of the authors and do not necessarily represent those of their affiliated organizations, or those of the publisher, the editors and the reviewers. Any product that may be evaluated in this article, or claim that may be made by its manufacturer, is not guaranteed or endorsed by the publisher.

## Supplementary Material

Supplementary Figure 1 Relationship between catheter derived wave reflection and arterial stiffness indices and RV systolic function. TAPSE was plotted against RC **(A)** and WS **(B)** obtained using catheter measurements, with separate regression lines for data acquired before challenge (Black solid line), during fluid challenge (Blue dashed line) and during dobutamine challenge (Red dotted line).

## Abbreviations

LAP: left atrial pressure
P: pressure
PA: pulmonary arterial
PAC: pulmonary artery compliance
PAP: pulmonary artery pressure
Pb: backward-traveling pressure
Pf: forward-traveling pressure
PH: pulmonary hypertension
PVR: pulmonary vascular resistance
RAP: right atrial pressure
RC: reflection coefficient
RV: right ventricle
RVOT: right ventricular outflow tract
TAPSE: tricuspid annular plane systolic excursion
TR: tricuspid valve regurgitation
U: velocity
WS: wave speed.
