# Supplementary figures and images for: Estimation of Pulmonary Arterial Wave Reflection by Echo-Doppler: A Preliminary Study in Dogs With Experimentally-Induced Acute Pulmonary Embolism
Source: Front Physiol. 2021 Dec 8;12:752550. doi: 10.3389/fphys.2021.752550 (PMC8692872; doi:10.3389/fphys.2021.752550)

**A**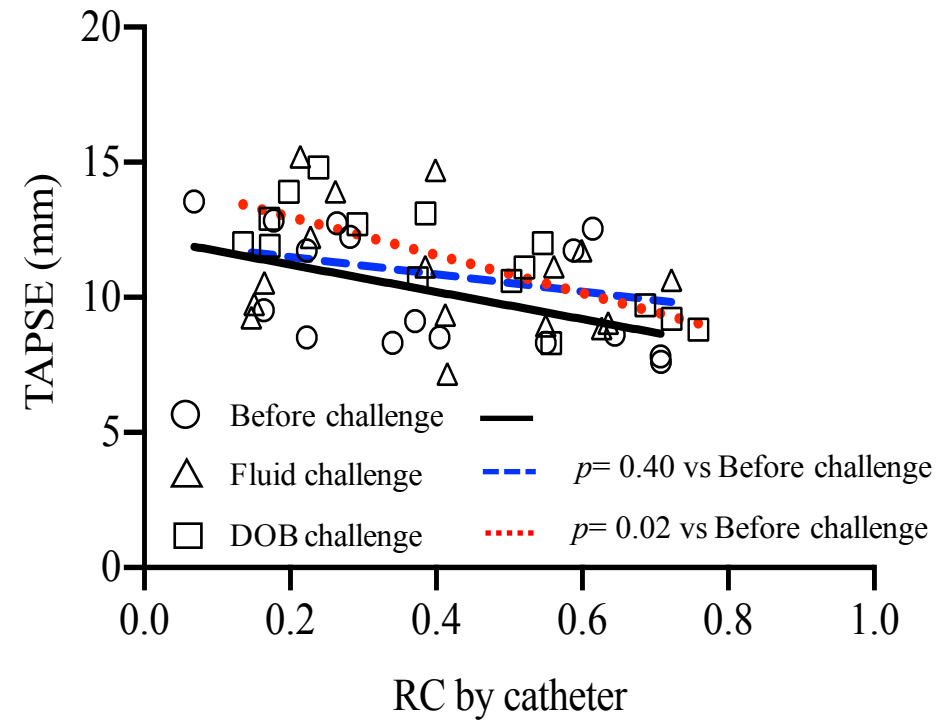**B**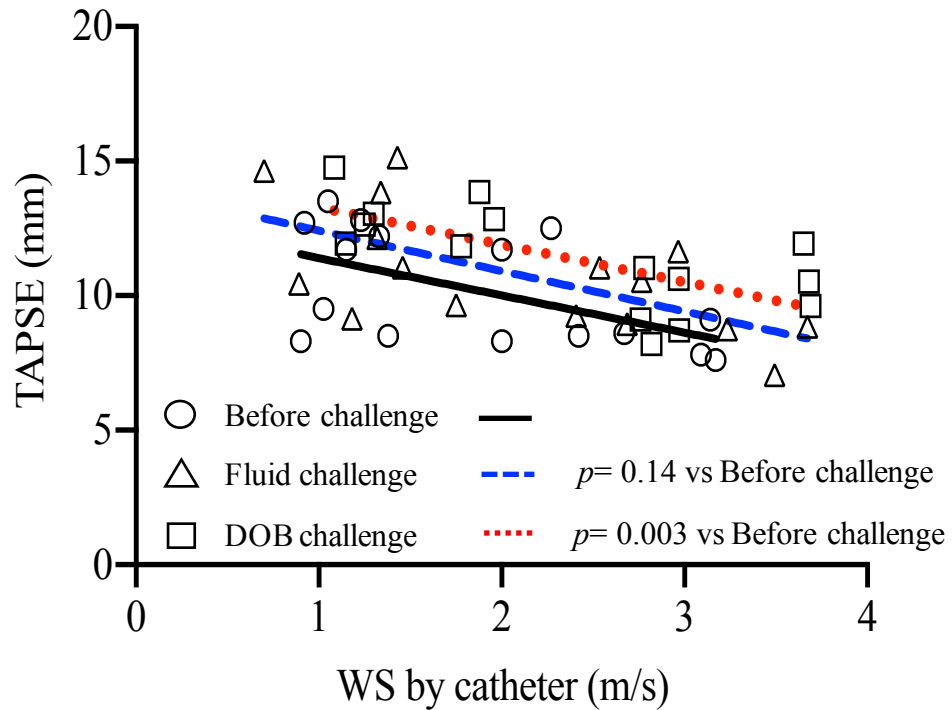

Supplement: Supplementary Figure 1 — Relationship between catheter derived wave reflection and arterial stiffness indices and RV systolic function. TAPSE was plotted against RC (A) and WS (B) obtained using catheter measurements, with separate regression lines for data acquired before challenge (Black solid line), during fluid challenge (Blue dashed line) and during dobutamine challenge (Red dotted line). [file Image_1.pdf]
