# Supplementary material for: Estimation of Pulmonary Arterial Wave Reflection by Echo-Doppler: A Preliminary Study in Dogs With Experimentally-Induced Acute Pulmonary Embolism
Source: Front Physiol. 2021 Dec 8;12:752550. doi: 10.3389/fphys.2021.752550 (PMC8692872; doi:10.3389/fphys.2021.752550)
Supplement: Supplementary file 2 [file Table_1.DOCX]

**Supplemental table 1. Effects of hemodynamic manipulations on catheter-derived wave reflection and arterial stiffness indices**

| **Fluid challenge** | | | | |
| --- | --- | --- | --- | --- |
|  | **Baseline** | | **Pulmonary hypertension** | |
|  | **Before** | **After** | **Before** | **After** |
| Pb, mmHg | 1.9±0.8 | 2.3±0.9 | 8.2±2.6 | 8.8±2.7 |
| Pf, mmHg | 8.6±1.5 | 9.9±2.0 | 14.6±3.5 | 15.5±3.0 |
| RC | 0.21±0.08 | 0.23±0.1 | 0.57±0.13 | 0.55±0.10 |
| WS, m/s | 1.1±0.2 | 1.2±0.3 | 2.6±0.5 | 3.0±0.5 |
| **Dobutamine challenge** | | | | |
|  | **Baseline** | | **Pulmonary hypertension** | |
|  | **Before** | **After** | **Before** | **After** |
| Pb, mmHg | 2.1±1.2 | 2.3±0.8 | 8.4±2.9 | 12.4±2.8* |
| Pf, mmHg | 8.2±1.0 | 10.5±2.4 | 16.6±2.1 | 22.6±8.1 |
| RC | 0.24±0.1 | 0.22±0.09 | 0.56±0.12 | 0.58±0.13 |
| WS, m/s | 1.4±0.2 | 1.5±0.3 | 2.45±0.6 | 3.2±0.4 |

Abbreviations: Pb, backward pressure; Pf, forward pressure; RC, reflection coefficient; WS, wave speed; * denotes p value < 0.05 vs before challenge
