# Supplementary material for: Estimation of Pulmonary Arterial Wave Reflection by Echo-Doppler: A Preliminary Study in Dogs With Experimentally-Induced Acute Pulmonary Embolism
Source: Front Physiol. 2021 Dec 8;12:752550. doi: 10.3389/fphys.2021.752550 (PMC8692872; doi:10.3389/fphys.2021.752550)
Supplement: Supplementary file 3 [file Table_2.DOCX]

Supplemental table 2. Hemodynamic determinants of catheter-derived wave reflection indices

|  | RC | | WS | |
| --- | --- | --- | --- | --- |
|  | R | P value | R | P value |
| PVR | 0.70 | <0.001 | 0.71 | <0.001 |
| PAC | -0.70 | <0.001 | -0.79 | <0.001 |
| CO | -0.61 | <0.001 | -0.55 | <0.001 |
| HR | -0.02 | 0.89 | 0.17 | 0.23 |
| sPAP | 0.62 | <0.001 | 0.73 | <0.001 |
| mPAP | 0.55 | <0.001 | 0.64 | <0.001 |
| mLAP | -0.13 | 0.36 | -0.05 | 0.74 |

Abbreviations: RC, reflection coefficient; WS, wave speed; PVR, pulmonary vascular resistance; PAC, pulmonary arterial compliance; CO, cardiac output; SV, stroke volume; HR, heart rate; sPAP, systolic pulmonary arterial pressure; dPAP, diastolic pulmonary arterial pressure; mPAP, mean pulmonary arterial pressure; mLAP, mean left atrial pressure.
